# Effects of OsomeFood Clean Label plant-based meals on the gut microbiome

**DOI:** 10.1186/s12866-023-02822-z

**Published:** 2023-03-30

**Authors:** Dwiyanto Jacky, Chia Bibi, Look Melvin Chee Meng, Fong Jason, Tan Gwendoline, Lim Jeremy, Chong Chun Wie

**Affiliations:** 1 Department of Medical Microbiology, Faculty of Medicine, University of Malaya, 50603 Kuala Lumpur, Malaysia; 2 AMILI, Singapore, 118261 Singapore; 3 PanAsia Surgery Pte Ltd, Singapore, 228510 Singapore; 4 OsomeFood, Singapore, 118494 Singapore; 5 School of Pharmacy, Monash University Malaysia, 47500 Subang Jaya, Malaysia

**Keywords:** Metagenomic, Microbiota, Nutrition, Intervention

## Abstract

**Background:**

Plant-based diets offer more beneficial microbes and can modulate gut microbiomes to improve human health. We evaluated the effects of the plant-based OsomeFood Clean Label meal range (‘AWE’ diet), on the human gut microbiome.

**Methods:**

Over 21 days, ten healthy participants consumed OsomeFood meals for five consecutive weekday lunches and dinners and resumed their regular diets for other days/meals. On follow-up days, participants completed questionnaires to record satiety, energy and health, and provided stool samples. To document microbiome variations and identify associations, species and functional pathway annotations were analyzed by shotgun sequencing. Shannon diversity and regular diet calorie intake subsets were also assessed.

**Results:**

Overweight participants gained more species and functional pathway diversity than normal BMI participants. Nineteen disease-associated species were suppressed in moderate-responders without gaining diversity, and in strong-responders with diversity gains along with health-associated species. All participants reported improved short-chain fatty acids production, insulin and γ-aminobutyric acid signaling. Moreover, fullness correlated positively with *Bacteroides eggerthii*; energetic status with *B. uniformis*, *B. longum*, *Phascolarctobacterium succinatutens*, and *Eubacterium eligens*; healthy status with *Faecalibacterium prausnitzii*, *Prevotella CAG 5226*, *Roseburia hominis*, and *Roseburia sp. CAG 182*; and overall response with *E. eligens* and *Corprococcus eutactus*. Fiber consumption was negatively associated with pathogenic species.

**Conclusion:**

Although the AWE diet was consumed for only five days a week, all participants, especially overweight ones, experienced improved fullness, health status, energy and overall responses. The AWE diet benefits all individuals, especially those of higher BMI or low-fiber consumption.

**Supplementary Information:**

The online version contains supplementary material available at 10.1186/s12866-023-02822-z.

## Introduction

The human gut microbiota comprises a vast and complex community of almost 100 trillion microorganisms (predominantly bacteria) and an estimated 5000 species [1] that inhabit the gastrointestinal tract. A normal gut flora, or core microbiota, consists primarily of Bacteroidetes (*Bacteroides* and *Prevotella*) and Firmicutes (*Clostridium*, *Enterococcus*, *Lactobacillus*, *Ruminococcus*, *Eubacterium* and *Faecalibacterium*). In normal, healthy adult guts, Firmicutes are the most abundant phyla, followed by Bacteroidetes [2]. However, the microbiome is dynamic, and various factors, including diet, age, lifestyle (e.g., stress), weight and the presence of disease, can affect its short-term and long-term diversity, composition and function. Changes to human health and wellbeing [3] have occurred when altering the diet with prebiotics and probiotics [4, 5] to modulate the microbiome, although evidence of this effect are mixed, and a complex relationship exists with the gut resistome [6]. While probiotics and prebiotics were not beneficial [7] in pre-diabetic adults and less beneficial than synbiotics in ulcerative colitis [8], probiotics were only marginally beneficial in diabetic patients [9], positively beneficial in cognitive impairment and mood or depressive disorders [10, 11], but negatively beneficial [12, 13] following antibiotic use. Thus, the potential for microbiome modulation through direct intervention is still actively pursued, as shown by the increase in randomized controlled trials being conducted.

Human gastrointestinal microbiota ferment intestinal mucus and indigestible dietary fiber, resulting in metabolites like bacteriocins, short-chain fatty acids (SCFAs), amino acids and vitamins [14]. These metabolites have key roles in activating intestinal immune responses to invading pathogens, while SCFAs also function as signaling molecules that regulate physiological processes like metabolism and inflammation [15, 16].

Plant-based diets are often linked to lower mortality rates [17], and their increased fibre and polyphenol levels are associated with a greater diversity in beneficial or healthy-gut microbes [18]. Plant-based diets also increase SCFA levels as they increase the amounts of microbes which metabolize complex carbohydrates and polysaccharides [19]. Omnivorous, ovo-lacto vegetarian, and vegan diets provide more nutrients that support a diverse gut microbiome, with the microbiome profile of vegans and vegetarians likely to have more beneficial bacteria than that of omnivores. In fact, omnivores have a more altered gut microbiome than vegans, as they have more bile-resistant microbes which can potentially become harmful [20]. One cross-sectional study found that Firmicutes and Bacteroidetes comprised up to 97.7% of the total vegan and omnivore gut microbiome, while Firmicutes comprised up to 58.6% and Bacteroidetes comprised 39% of the microbiome [21]. Interestingly, *Prevotella* species dominate in populations with plant-based diets, like those in Africa, Asia, and South America, whereas *Bacteroides* dominate in Western populations with diets high in animal proteins and saturated fats [22]. Individuals with diets rich in indigestible carbohydrates like whole grains and wheat bran have more *Bifidobacterium* and *Lactobacillium* while those with diets high in starches and whole grain barley may have more lactic acid bacteria (LAB; e.g., *Lactobacillus sp.*).

How the gut microbiome composition and function might be changed by short-term dietary changes remains to be established. Diet impacts the microbiome and may produce a chronic but mild inflammation, leading to chronic diseases like type II diabetes, cardiovascular disease and cancer, or chronic conditions like obesity [23]. Unregulated changes or imbalances in microbiome composition or function [24] can also manifest clinically as rheumatic diseases [25], psychiatric disorders [26], diabetes [27], hypertension [28] and cancers [29]. Gut microbiome manipulation offers a way to improve these disease risks. One dietary intervention study found that microbiome changes caused by switching diets [30], with *Prevotella*-enriched vegetarians or *Bacteroides*-enriched Western diet individuals all experiencing altered microbiome compositions within 24 h of swapping diets [30, 31]. A study in Thai vegetarians [32] also found similar results with *Prevotella*-enriched microbiomes in vegetarians. However, microbiome composition requires further study as others have noted conflicting results [33].

The commercially-available AWE by OsomeFood™ [34], is a nutrition-focused, both clean and functional plant-based meals that combines its OsomeFood super ingredients and its iteration of different clean sauces and ingredients to complete a meal. All meals are made without artificial additives, extracts, fortifications, synthetic ingredients, genetically-modified organisms or preservatives and yet naturally supercharged with nutritional goodness. All ingredients undergone strict qualifications, from the source of produce, cleansing technologies, activation through dehydrating or fermentation, to encapsulation and curated pairing of nutrients to achieve optimal absorption and maximum nutrition. OsomeFood’s food is made primarily from fungi and algae (single cell protein) as well as nuts and plant protein.

## Methods

The AWE study aimed to assess changes in wellbeing and the gut microbiome signature in ten study participants (*n* = 10) who consumed 900–2000 cal/week plant-based meals provided by OsomeFood. Participants were able to continue their regular diets for all meals except weekday lunches and dinners. For 21 days, participants had access to over 30 different types of meals. Some examples of OsomeFood meals are fish balls, fish cakes, protein noodles and collagen egg made from fungi (including mushrooms fermented into mycoproteins), Undaria pinnatifida seaweed, white chia seeds, burdock root and kombu kelp seaweed. [35]. To prepare the meals, participants are only required to thaw each OsomeFood meal pack and consume them heated up with the recommended heating methods.

### Study protocol and design

**Fig. 1:**
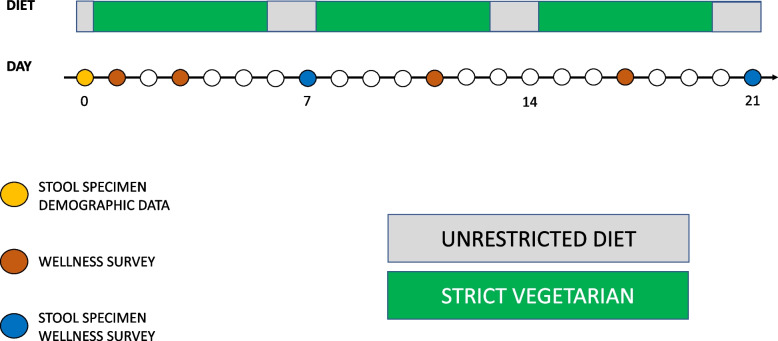
Schematic of study design. Subjects underwent a 21-day non-continuous plant-based diet intervention, with stool specimen collected on day (D)0, D7, and D21. Meanwhile, wellness survey was administered on D1, D3, D7, D11, D17, and D21. Demographic data were obtained on D0

This study has been approved by the AMILI Institutional Review Board, which adheres to the Declaration of Helsinki (AMILI IRB Ref: 2022/0201). All participants were at least 18 years of age and have provided their written informed consent. Participants strictly adhered to OSomeFoods’ plant-based meal plans for five consecutive days (Monday through to Friday; ‘AWE’), and were allowed unrestricted meals for two consecutive days over the weekend (Saturday and Sunday; ‘non-AWE’) (Fig. 1). The study was performed in March 2022. Healthy participants were recruited from Singapore, all of whom provided written informed consents for their participation in this research study.

### Participants/inclusion and exclusion criteria

Included participants were those aged 21 years and older, with a Body Mass Index (BMI) between 18 and 28, able to provide informed consent, and who were meat eaters. Excluded participants were those using oral antibiotics, antifungal and/or antiviral treatments within the prior 3 months, those with existing medical issues, those on any other long-term medications, and vegetarians.

### Data and sample collection

Participants were not required to complete and maintain a food frequency questionnaire (FFQ) on AWE days but did self-report their meal consumption on non-AWE days. On AWE days, subjects were followed-up on days (D) 1, 3, 7, 11, 17, and 21, to record their self-perception of three metrics: satiety, energy, and health. Insights from two prior in-house food trial pilot studies showed that changes in gut microbiome diversity and abundance occurred by 21 days (data not shown), hence this was used as our observational timepoint. Each metric was evaluated on a 3-point ordinal scale (1: worst rating, 3: best rating). For microbiome analysis, participants provided stool samples on the first day of the intervention (D1), and at the end of week 1 (D7) and week 3 (D21).

### Data and sample analysis

To document microbiome variations occurring during the AWE diet period, and identify associations with the additional FFQ, we analyzed species and functional pathway annotations, Shannon diversity and cheat day calorie intake subsets. For species and functional pathway annotation, DNA was extracted from stool samples using the QIAamp® PowerFecal® Pro DNA Kit Handbook (QIAGEN GmbH, Hilden, Germany) according to the manufacturer’s protocols, and was processed for shotgun sequencing using the Illumina NovaSeq 6000 Sequencing System (RRID:SCR_016387; Illumina, San Diego, CA, USA) according to the manufacturer’s protocols.

### Species and functional pathway annotations

DNA was extracted from the collected stool samples and shotgun sequencing was performed by Macrogen Asia Pacific Pte. Ltd. (Singapore). The resulting FASTQ sequences were then fed into the BioBakery 3 pipeline for reference-based taxonomic pathway annotation using MetaPhlAn 3 and functional pathway annotation using HUMAnN.

### Statistical analysis

After excluding species with < 1% relative abundance and prevalence in < 5% of the participants, 236 species remained for further analysis. We determined whether the change in diet affected microbiome composition across the study duration through permutational multivariate analysis of variance analysis (PERMANOVA). Briefly, Shannon diversity was calculated using the R package phyloseq version 1.40.0. Beta-diversity analysis was conducted on the species and pathway composition data which was centered-log-ratio transformed to account for the compositionality of the dataset. Subsequently, features with < 1% abundance and < 5% prevalence were excluded. PERMANOVA was conducted to determine the significant variation across the samples using the R package vegan version 2.6–2 using the Euclidean distance with 999 permutations. Differentially-abundant features were determined using pairwise Wilcoxon Rank Sum test, with multigroup comparisons adjusted using the Benjamini–Hochberg method. Questionnaire output and feature abundance were correlated using a linear mixed model method under the R package lme4 version 1.1–29, with age, sex, and BMI accounted as fixed effect and subject adjusted as random effects.

## Results

### Participants demographics

Ten participants (50% male, 50% female) were recruited and completed the study, and ranged in age from 20-49 years, although the majority were aged 30-39 years. The majority (70%) were also overweight [BMI: 23-24.9kg/m^2^], while the remaining participants were either underweight (10%), normal (10%) or obese (10%).

### Species composition analysis

**Fig. 2:**
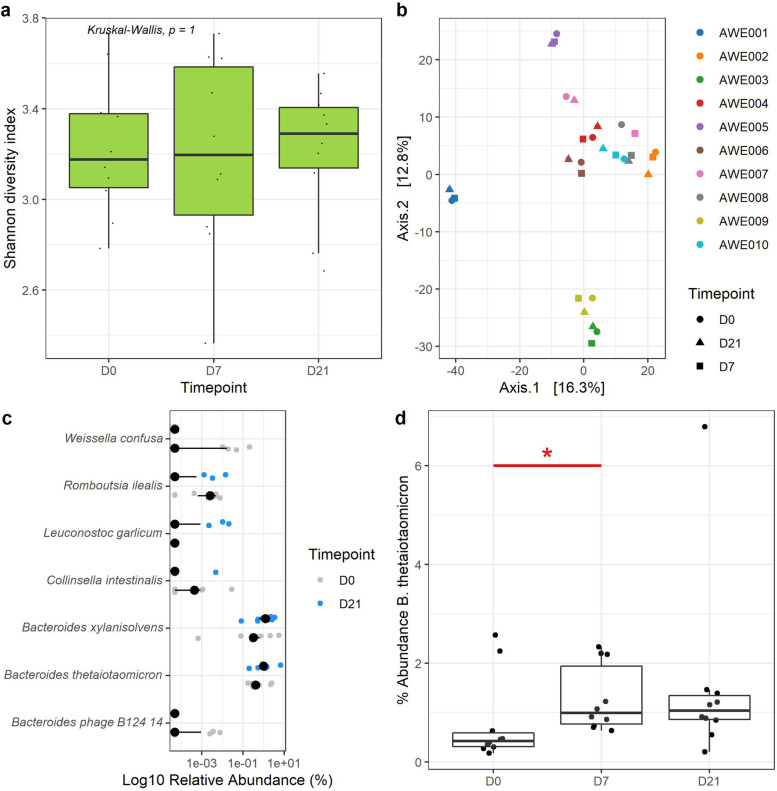
**a** Shannon diversity index of the subjects over the intervention period; **b** Principal component analysis of the subjects’ gut microbiota profile ordinated based on subject (color) and timepoint (shape) using centered-log-ratio transformation on a Euclidean distance, with significant variation across timepoint (Stratified PERMANOVA R2 = 0.022, *p* = 0.008); **c** Species differentially abundant between baseline and day 21 (end of the intervention period), measured using Wilcoxon test and adjusted for multiple comparison using the Benjamini–Hochberg method (q < 0.1); **d** Abundance of *B. thetaiotaomicron* across timepoint

A total of 369 species were detected from the compiled gut microbiome profile; 236 of which remained after excluding those with < 1% relative abundance and found in < 5% subjects. The Shannon diversity metric is commonly used to assess the diversity of microbial communities in human gut microbiome studies. The Shannon diversity index accounts for the number of different types of microorganisms (species richness) and their relative abundances and provides a more comprehensive measure of diversity than metrics that consider only one of these factors. In human gut microbiome research, a high Shannon diversity (a diverse gut microbiome) is generally considered to be a marker of gut health. Conversely, low microbial diversity has been associated with a variety of health conditions, including inflammatory bowel disease, obesity, and type 2 diabetes. There was no significant variation in Shannon diversity across timepoint (KW test, *p* > 0.05; Fig. 2a), even when the analyses were done across demographic factors (Supplementary Fig. 1). Despite this, beta-diversity analysis determined a significant variation in the species composition across timepoint (PERMANOVA stratified for subject variation, permutations = 999, *R*^2^ = 0.0222, Pseudo-F = 0.3069, *p* = 0.005; Fig. 2b). Differential abundance analysis identified seven species with different abundances between D0 and D21, three of which (*Bacteroides thetaiotaomicron*, *Bacteroides xylanisolvens* and *Leuconostoc garlicum*) were elevated at the end of the study, while four were depleted (Wilcoxon test, q < 0.1; *Weissella confusa*, *Romboutsia ilealis*, *Collinsella intestinalis* and a *Bacteroides* phage) (Fig. 2c). Among these, *B. thetaiotaomicron* was significantly higher in D7 and D21 compared to the baseline (Wilcoxon test, *p* < 0.05; Fig. 2d). Additionally, 19 bacteria species related to cancer, inflammation, sepsis, weight management and non-alcoholic fatty liver disease (NAFLD), were suppressed throughout and at the end (D21) of the study in participants, with a constant increase in abundance in 30 species known to confer health benefits such as cholesterol, immunity and weight management (Supplementary Fig. 2).

### Correlations with functional pathway data

**Fig. 3:**
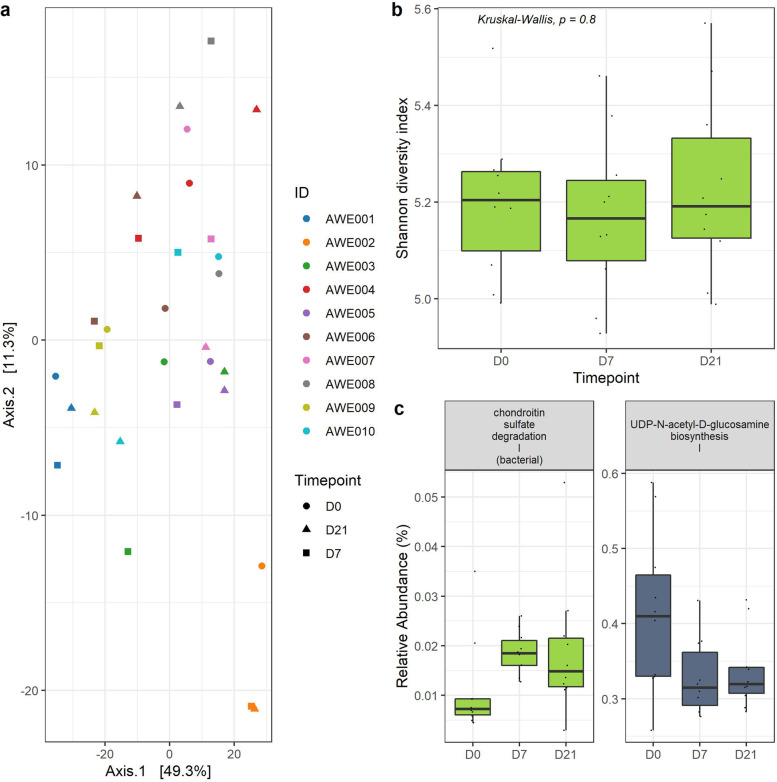
**a** Principal component analysis of the subjects’ gut functional pathway composition profile ordinated based on subject (color) and timepoint (shape) using centered-log-ratio transformation on a Euclidean distance, with nonsignificant variation across timepoint (Stratified PERMANOVA R2 = 0.037, *p* = 0.204); **b** Shannon diversity of the functional pathway profile across timepoint; **c** Abundance of chondroitin sulfate degradation I and UDP-N-acetyl-glucosamine biosynthesis I across timepoint

A total of 453 functional pathways were found, out of which 384 remained after excluding low-abundance (< 1%) and low-prevalence pathways (< 5%). Similar to the species composition profile, Shannon diversity analysis identified no significant difference across timepoints (KW test, *p* > 0.05; Fig. 3b). However, PERMANOVA also failed to identify any significant difference across timepoints based on composition profiles (PERMANOVA stratified for subject variation, permutations = 999, *R*^2^ = 0.037, *p* > 0.05) (Fig. 3a). Despite this, pairwise comparison across timepoints identified two functional pathways with significantly different abundance: UDP-N-acetyl-D-glucosamine biosynthesis I and chondroitin sulfate degradation I (bacterial) (Wilcoxon test, q < 0.05, Fig. 3c). Moreover, all participants reported improved SCFA production, insulin and γ-aminobutyric acid (GABA) signaling after the AWE diet, with related functional pathways continuing to increase and associated with participants’ improved health profiles (Supplementary Fig. 3). Most of the functional pathways that improved after the AWE diet were associated with vitamin K production, immunity, gut lining integrity and detoxification.

### Microbiome correlation with wellbeing Survey

**Fig. 4:**
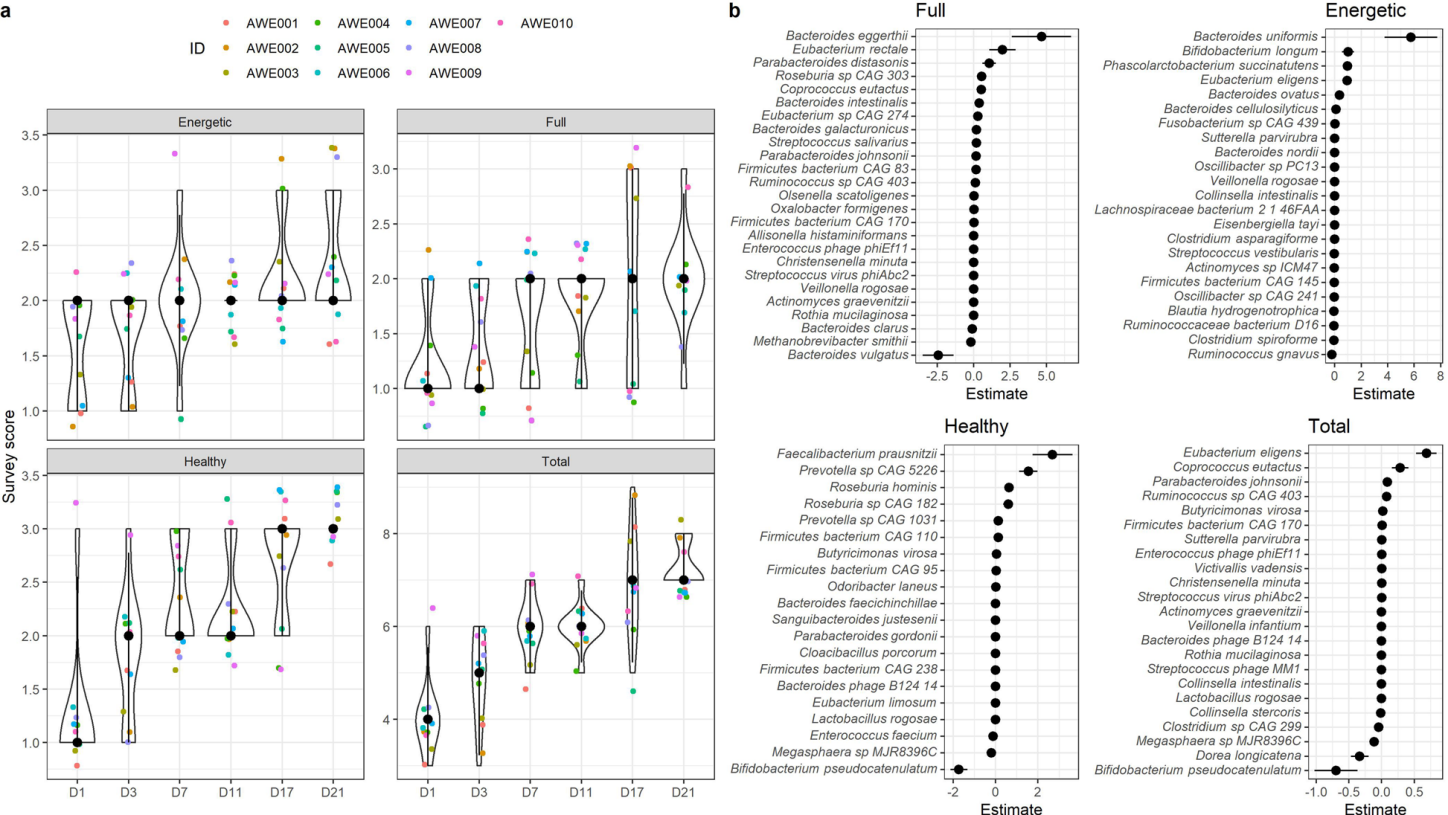
**a** Three-point self-rate survey on energy (energetic), satiety (fullness), and health (healthy), and total (Total) level of the subjects throughout the intervention period; **b** Species significantly associated with each of the survey variables, analysed using linear mixed model, adjusted for age, sex, and BMI, with statistical significance measured using the likelihood ratio test (*p* < 0.05)

Participants’ reported outcome measures of general wellbeing were evaluated through a weekly (D7, 14 and 21), 3-point survey of whether they felt they had more energy (‘energetic’), fullness (meal satiety), and perceived healthiness, throughout the study duration (at 6 evaluation timepoints). Overall, and as the study progressed, all participants’ scores of meal satisfaction, energy levels and feeling healthier, increased (linear model *p* < 0.05, Fig. 4a). Importantly, several species were significantly correlated with the participants’ survey metrics (Fig. 4b). Positive correlations were seen for fullness with *Bacteroides eggerthii*; energetic status with *B. uniformis*, *B. longum*, *Phascolarctobacterium succinatutens*, and *Eubacterium eligens*; healthy status with *Faecalibacterium prausnitzii*, *Prevotella sp. CAG 5226*, *Roseburia hominis*, and *Roseburia sp. CAG 182*; and overall response with *E. eligens* and *Corprococcus eutactus*. Negative correlations were seen for fullness with *B. vulgatus*; for healthy status with *Bifidobacterium pseudocatenulatum*; and overall response with *Dorea longicatena* and *B. pseudocatenulatum*.

### Effect of BMI on microbiome changes

**Fig. 5:**
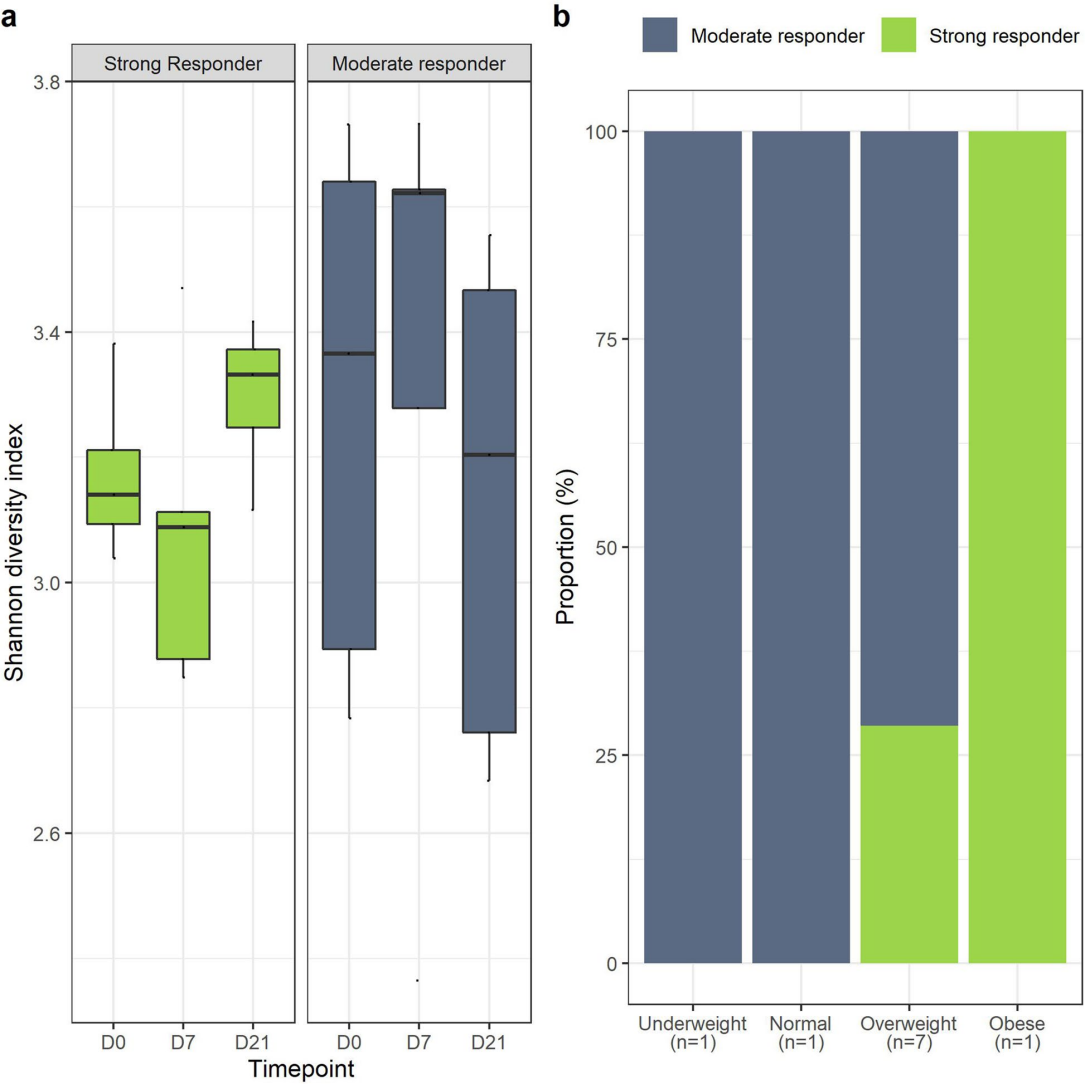
**a** Distribution of responders across subject’s bodyweight class; **b** Shannon diversity of subjects across responder categories

Participants were classified as either moderate or strong responders based on the net Shannon diversity change after the three weeks intervention period (Fig. 5a). Interestingly, strong responders all belonged to a higher weight category compared to moderate responders (Fig. 5b).

### Microbiome correlation with nutritional intake

Data from FFQ completed during the non-AWE phase were divided into a lower and upper quartile for convenience, and analyzed to determine if changes in participants’ microbiome diversity were nutrition-related. No nutrient consumption metric was significantly associated with changes in microbiome diversity (Wilcoxon test, *p* > 0.05; Supplementary Fig. 4). Despite this, subjects with low fibre consumption during the non-AWE phase seemed to exhibit a higher microbiome diversity than those with higher fibre consumption.

We evaluated whether nutrient consumption was correlated with species that are also known to be beneficial or pathogenic and found 42 beneficial and 17 pathogenic species in our participants (Table 1).

**Table 1:** Number of known beneficial and pathogenic species observed in our cohort

Status	Presence	N
Beneficial	Absent	8
Beneficial	Present	42
Pathogenic	Absent	1
Pathogenic	Present	17

Nutritional values from the FFQ data was correlated with these 59 species, filtered to include species-nutrient pairing with *p* < 0.05 and absolution R2 > 0.4. Nineteen unique species (pathogenic, *n* = 7; and beneficial, *n* = 12) were found, and associated with five nutrient metrics (Supplementary Fig. 5). Beneficial species were generally negatively associated with calorie, carbohydrate, fat, and protein intake, except for *P. faecium* which had a mixed outcome for fibre intake. In contrast, pathogenic species demonstrated the opposite trend as nutrients positively correlated with bacterial abundance, except for *D. longicatena*. Fibre consumption was also negatively associated with all species associated with pathogenic features.

## Discussion

### Overview

We found 369 species and 453 functional pathways in our participants’ gut microbiomes across the study duration, with diversity remaining stable and unaffected by demographics, although overweight participants had more diversity at the end of the study (day 21) than those with normal BMI. All overweight participants responded better than other weight groups to the AWE diet. Additionally, participant-reported wellbeing scores (satiety, energetic and healthier) were unanimously higher at day 21, potentially due to diet-associated improvements in SCFA production, insulin signaling and GABA signaling. On AWE diet days, exclusion of animal fat and lower protein consumption likely created a dietary composition that suppressed 19 disease-related bacteria, as others have reported [36, 37].

### AWE diet induced species changes in the gut microbiome

In the human distal gut, *B. thetaiotaomicron* ferments simple carbohydrates and complex plant polysaccharides [38]. In mice, *B. thetaiotaomicron* BPI-5482 significantly increased total body fat and promoted fat storage [39]. *B. thetaiotaomicron* increases the hepcidin hormone [40] which can worsen metabolic disorders, increase weight gain and fasting glucose levels, impair glucose tolerance and increase liver accumulation of fatty acids. However, colon fermentation by *B. thetaiotaomicron*, *B. eggerthii* and *B. xylanisolvens*, produces beneficial prebiotic metabolites in obese individuals and obesity-related conditions [41]. *B. xylanisolvens* also ferments alginates [42] into SCFAs that fuel intestinal epithelial and immune cells [43], maintain gut health [44] and inhibit large intestine production of toxic metabolites [42].

*Leuconostoc garlicum* exists naturally in fruits, vegetables and plant roots [45], dairy products, wine and sugar [46], but are not typically part of human gut flora [47]. *L. garlicum* ferments sucrose into dextran [48] and is often used as a probiotic or starter [45] in plant-based fermented foods like kimchi. The increased abundance of *L. garlicum* is therefore expected, considering the plant-based nature of the AWE diet. However, more work is needed to understand the probiotic roles of the *Leuconostoc* genus.

The *W. confusa* F213 [49]strain ferments glucose into lactic acid, ethanol and/or acetate in fermented foods [50]. However, it leads to continuous ethanol [51] production in the large bowel, thus affecting peripheral blood alcohol levels. In rats, [51] hyperlipidemia and NAFLD resulted from high-fructose intake elevating ethanol levels in faeces and peripheral blood. The suppression of W. confuse following AWE diet intervention therefore shows the potential to confer protective effect against these conditions.

*Romboutsia ilealis* [52] is linked to protective human leukocyte antigen (HLA) haplotypes, although some HLA allele combinations and gut microbiome changes are associated with autoimmune diseases like type 1 diabetes [53]. Probiotic consumption increases *R. ilealis* levels [54] and decreases pro-inflammatory plasma cytokines. In primary sclerosing cholangitis with inflammatory bowel disease, gluten-free diets reduced *R. ilealis* [55]. Similarly, in mice studies [56] of colitis, selenium-enriched Lactobacillus acidophilus improved *Romboutsia*-promoted intestinal inflammation and significantly reduced their levels.

*Collinsella intestinalis* ferment carbohydrates but not fiber, and flourish with low-fiber diets [57] where they may alter gut microbiome fermentation and cause harmful metabolic or inflammatory effects. High *Collinsella* levels are also associated with westernized [58], low-fibre and high red meat diets [59], with chronic diseases [60] and negative effects on cholesterol metabolism [61]. *Collinsella* levels are decreased by high-fibre diets [64]. After six weeks in one low-calorie weight-loss program, *Collinsella* significantly decreased by 8.4-fold [62] yet weight-loss persisted along with fecal microbiome changes. *Collinsella* facilitate intestinal absorption of cholesterol (thus increasing circulating cholesterols) [63], reduce liver glycogenesis and increase triglyceride production. In high-fibre macrobiotic diets, *Collinsella* improve metabolic responses in type II diabetes [64], although *Collinsella* can also be present at high levels [1].

Bacteriophages eliminate bacteria selectively [65], and can impact their host’s metabolism and immunity [66]. Dietary changes may increase stress in bacterial hosts, increase active phage numbers, and cause lysogenic phages to enter lytic stages [67].

### Changes in functional pathways due to the AWE diet

Changes in species abundance may not correlate with changes in function, as we detected few significant differences from the detected 384 functional pathways across timepoints. Nevertheless, our pairwise comparison identified UDP-N-acetyl-D-glucosamine biosynthesis I and chondroitin sulfate degradation I (bacterial) to be reduced and elevated at the end of the intervention period, respectively. UDP-N-acetyl-D-glucosamine is found in barley plant extracts and mung-bean seedlings [68], where it is part of a glucose metabolism pathway that is increased in insulin-resistant obese Chinese children and adolescents [69]. It may also play roles in the emergence of insulin resistance and diabetic vascular complications [70]. Chondroitin sulfate degradation was thought to be depleted in in vitro studies examining the effect of consuming a Korean traditional fermented soybean soup [71].

### Wellbeing surveys correlated with various gut microbiome species

Several species was positively linked with the wellbeing survey administered to the subjects across the intervention. Fullness was significantly positively correlated with *B. eggerthii* and negatively with *B. vulgatus*; energetic status was significantly positively correlated with *B. uniformis*, *B. longum*, *P. succinatutens*, and *E. eligens*; healthy status was significantly positively correlated with *F. prausnitzii*, *Prevotella sp.* CAG 5226, *Roseburia hominis*, and *Roseburia sp.* CAG 182, but negatively with *B. pseudocatenulatum*. The overall response was significantly positively correlated with *E. eligens* and *C. eutactus* and negatively with *D. longicatena* and *B. pseudocatenulatum*.

*Bifidobacterium* may protect against obesity, reduce serum cholesterol, triglyceride, and glucose levels, and improve insulin resistance and glucose tolerance [72]. *B. pseudocatenulatum* degrades xylan-derived carbohydrates into SCFA [73] and may improve colitis by modifying the gut microbiome, blocking inflammatory cytokines and signaling, and maintaining the intestinal barrier [74]. Our study negatively correlated *B. pseudocatenulatum* with health status, but more work is needed to determine if this was a strain-specific observation.

*C. eutactus* is an obligate anaerobe that and is a constituent of healthy guts [75] but is present at the lowest abundance in the irritable bowel syndrome D subtype [76]. *C. eutactus* leads to the production of butyrate [77], while *Coprococcus* species are generally associated with a better quality of life and are depleted in depression [78]. Another obligate anaerobe, *D. longicatena*, is negatively correlated with markers of dyslipidaemia or insulin resistance, indicating its beneficial probiotic effect [79]. However, its role is unclear as it is increased in Crohn’s remissions [80] but decreased in heart failure [81].

Supplementation with polyphenol-rich citrus fruit extracts has been found to significantly increase levels of *B. eggerthii* and *Roseburia sp.* [82], while red wine polyphenol and sorghum bran consumption supported *Roseburia sp.* growth in overweight participants. *Roseburia sp.* is abundant in the intestine where it produces butyrates, and may combat inflammation and obesity. Fecal sequencing detected more *Prevotella* and *Roseburia* but fewer Bacteroides in omnivores than in vegans and vegetarians [83], while abundant *Roseburia sp.* CAG182 was detected [84] in severe steatosis versus no-steatosis patients. *Roseburia* CAG182, *F. prausnitzii* and *E. eligens*, are part of a microbial signature for cardiometabolic health, and cluster with other species in healthy plant- or animal-based foods [85]. This agrees with our positive correlation of these species with energy, health and overall responses, even in our small cohort of overweight or obese individuals. Bacteroidetes associate positively with fat but negatively with Firmicutes. *B. eggerthii* and *B. uniformis* were enriched in individuals with low visceral fat [86], while *B. uniformis* protected against metabolic disorders and obesity [86]. Thus, some *Bacteroides* species could be considered as probiotics in the management of obesity.

We correlated several Firmicutes species with a healthy condition. *R. hominis* is known to upregulate genes for chemotaxis, mobilization and motility [87], and plays roles in gut barrier function and immune modulation. Additionally, *P. succinatutens* degrades dietary fibre into succinate and subsequently into propionate, thus conferring anti-inflammatory and antitumor properties [88]. It is worth noting that *P. succinatutens* has also been reported to be enriched in patients suffering from ulcerative colitis [89], warranting further study on its role in human health.

### Association between nutritional intake and the microbiome

Our participants’ nutrient consumption also correlated with seven and twelve species which have been associated with pathogenicity and beneficial health effect, respectively. Beneficial species were negatively associated with calorie, carbohydrate, fat and protein consumption, although the impact on P. faecium following fibre consumption was mixed. Pathogenic species positively correlated with bacterial abundance, and importantly, negatively correlated with fibre consumption. Commensal gut bacteria produce SCFAs by fermenting dietary fibre, which lowers postprandial insulin responses and blood glucose levels. Low-fiber diets support mucus-degrading bacteria and the growth of pathogens that compromise the colonic mucus barrier [90]. Dietary fibre also influences the immune system to produce more T cells which suppresses inflammation [3] and regulates the inflammasomes [91]. A short-term increase in dietary fiber can significantly increase *F. prausnitzii* [92], whereas consumption of apple pectin-derived and inulin-derived indigestible carbohydrates increase *B. uniformis*, *B. eggerthii*, *B. thetaiotaomicron* and *B. vulgatus* [93]. However, over the long-term, gut microbiome stability was similar between individuals fed high-fat/low-fiber or low-fat/high-fiber diets and those on identical, short-term diets, although *Bacteroides* and *Prevotella* were more associated with long-term consumption of proteins, carbohydrates and animal fats. Furthermore, short-term consumption of animal-based diets led to fewer Firmicutes, while plant-based diets increased bile-tolerant microbes, like *Bacteroides* [31]. Though limited, the existing human studies on specific foods show that microbes like Bacteroidetes are increased by fat intake, while microbes like Firmicutes decrease.

Vegan and omnivorous gut microbiomes are dominated by both Firmicutes and Bacteroides, while omnivores have more Proteobacteria and *Roseburia*/*Eubacterium rectal* [94], vegans have more Verrucomicrobiota, lacto-vegetarians have more *F. prausnitzii*, and vegetarians and vegans both have fewer *Bacteroides* or *Bifidobacterium * [95]. *F. prausnitzii* may protect against obesity as it produces butyrate and anti-inflammatory metabolites [96]. As *F. prausnitzii* was depleted in metabolically healthy but obese individuals, its absence may promote obesity. Additionally, *Prevotella* are positively correlated with high-fibre diets [97], and *Prevotella*-rich diets are linked to weight-loss [98], less cholesterol [99] and improved glucose and succinate metabolism [100]. A Mediterranean diet interventional study found that *Prevotella* degrades complex polysaccharides in high-fibre diets, reducing insulin resistance in overweight participants [101]. These observations supported the beneficial effect of AWE diet which observed the positive correlation between health status and Prevotella CAG:5226.

### Benefit of AWE diet on subjects in the high BMI ranges

The majority of our cohort were overweight or obese. Interestingly, obese and overweight patients were more likely to respond to the AWE intervention based on their Shannon diversity. Nevertheless, we acknowledge the skewed distribution of our data which had employed participants from a higher BMI group. Despite this, our observation suggests the potential effect of AWE diet in assisting weight management.

### Limitation

Our study was limited by a limited sample size and ethnic diversity, and inability to record macronutrient and micronutrient consumption which prevented correlations between nutrient consumption and diet intervention or microbiome effects. Further study is also needed to ascertain the long-term effects of the diet on the microbiome and confirm the persistence of the diet’s benefits.

## Conclusion

Our analysis of microbiome changes occurring during the consumption of the plant-based, AWE diet, highlighted the benefits of the increase in fibre intake, even though participants adhered to this meal plan for just 5 days a week and resumed their normal diets in for a subsequent 2 days. Participants, especially overweight and obese individuals, experienced positive changes associated with fullness, health status, energy and overall response. While more data is needed on the exact physiological impact exacted by the alteration of each microbe, our data suggest that the AWE diet benefits all individuals, especially those of higher BMI ranges.

## Supplementary Information

**Additional file 1: Supplementary Fig 1.** Demographic subset of Shannon diversity index across age, sex, and BMI. **Supplementary Fig 2.** Species with increasing abundance over the intervention period. **Supplementary Fig 3.** Pathways with increasing abundance over the intervention period. **Supplementary Fig 4.** Shannon diversity of subjects based on levels of nutrients consumed. **Supplementary Fig 5.** Correlation between reported beneficial and pathogenic species with nutrient consumed.

## Data Availability

The raw sequence data used in this study has been uploaded to NCBI under the BioProject number PRJNA939268.
