# Supplementary material for: Effects of OsomeFood Clean Label plant-based meals on the gut microbiome
Source: BMC Microbiol. 2023 Mar 30;23:88. doi: 10.1186/s12866-023-02822-z (PMC10061721; doi:10.1186/s12866-023-02822-z)
Supplement: Supplementary file 1 — Additional file 1: Supplementary Fig 1. Demographic subset of Shannon diversity index across age, sex, and BMI. Supplementary Fig 2. Species with increasing abundance over the intervention period. Supplementary Fig 3. Pathways with increasing abundance over the intervention period. Supplementary Fig 4. Shannon diversity of subjects based on levels of nutrients consumed. Supplementary Fig 5. Correlation between reported beneficial and pathogenic species with nutrient consumed. [file 12866_2023_2822_MOESM1_ESM.pdf]

	Title	Caption
1	Supplementary Fig 1	Demographic subset of Shannon diversity index across age, sex, and BMI
2	Supplementary Fig 2	Species with increasing abundance over the intervention period
3	Supplementary Fig 3	Pathways with increasing abundance over the intervention period
4	Supplementary Fig 4	Shannon diversity of subjects based on levels of nutrients consumed
5	Supplementary Fig 5	Correlation between reported beneficial and pathogenic species with nutrient consumed

Supplementary Figure 1

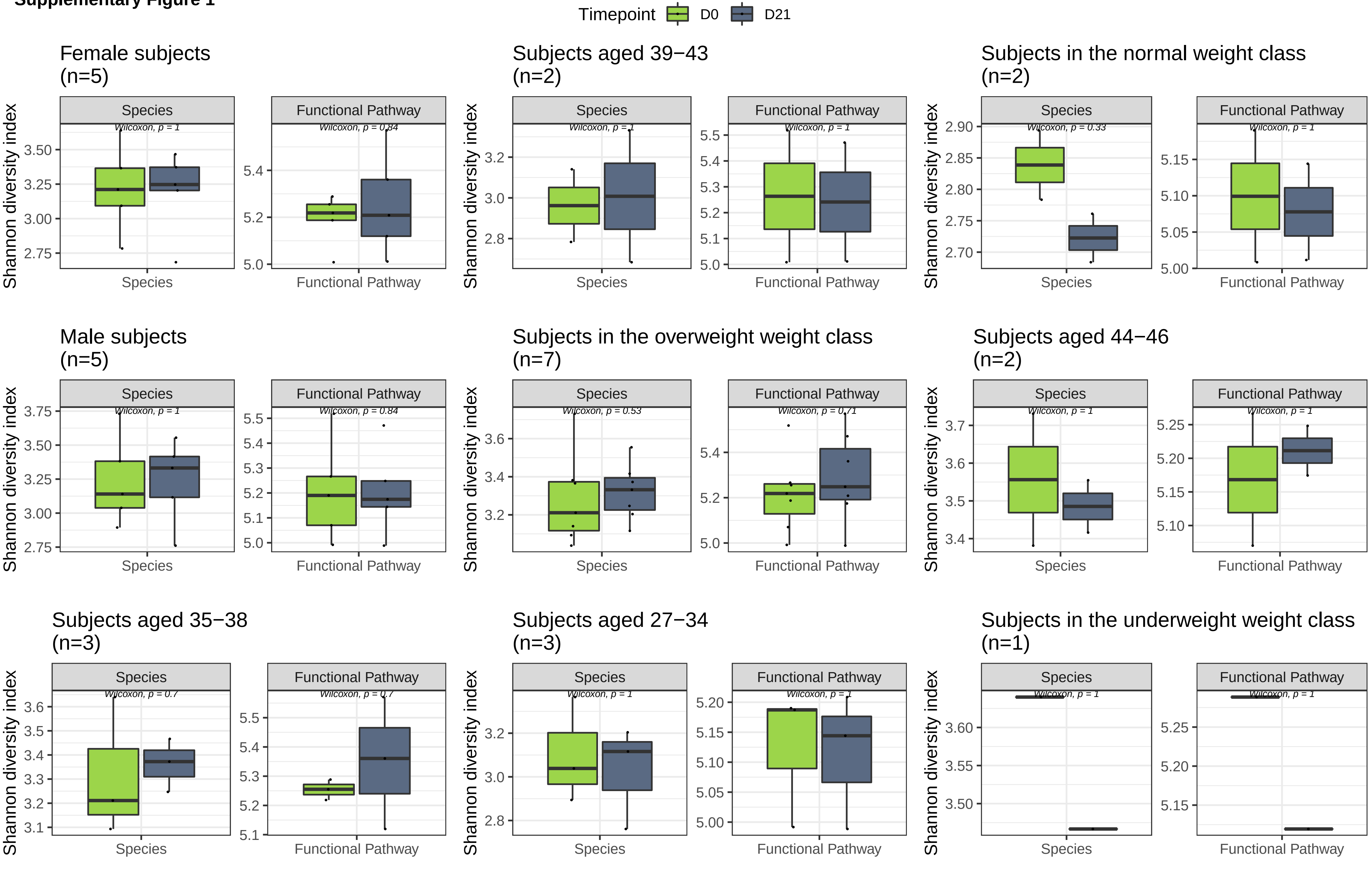

Supplementary Figure 1: Demographic subset of Shannon diversity index across age, sex, and BMI

Supplementary Figure 2

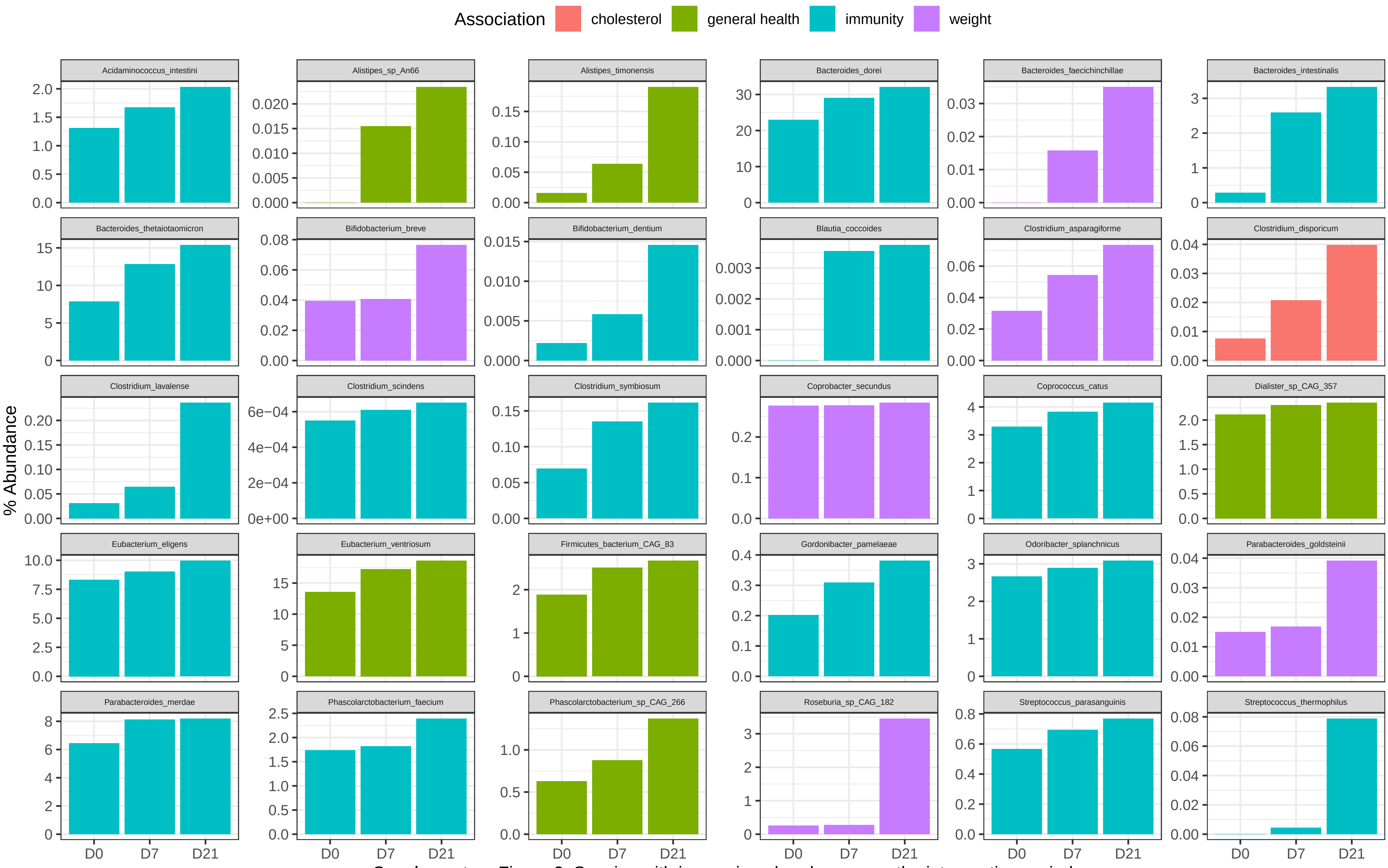

Supplementary Figure 2: Species with increasing abundance over the intervention period

Supplementary Figure 3

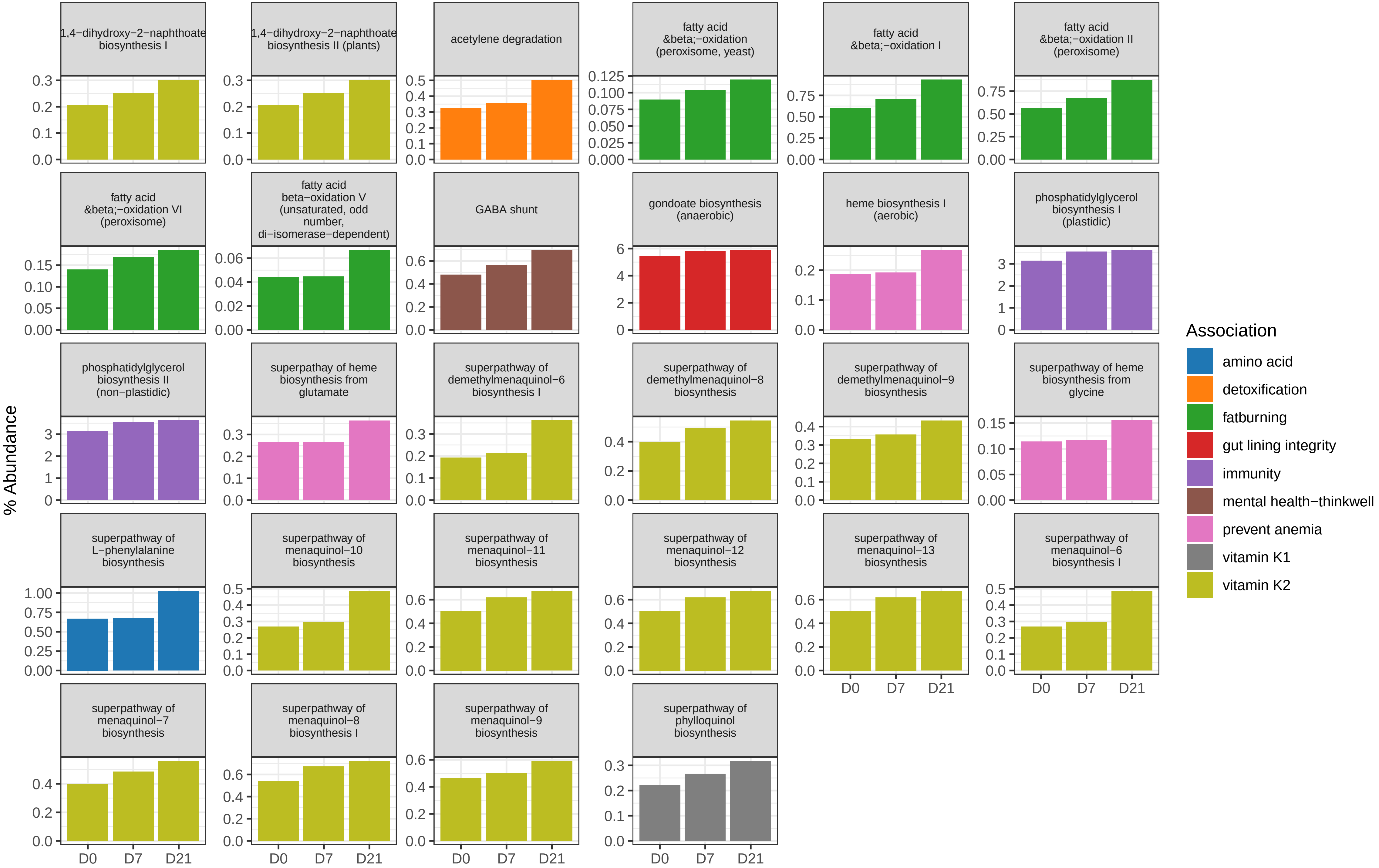

Supplementary Figure 3: Pathways with increasing abundance over the intervention period

Supplementary Figure 4

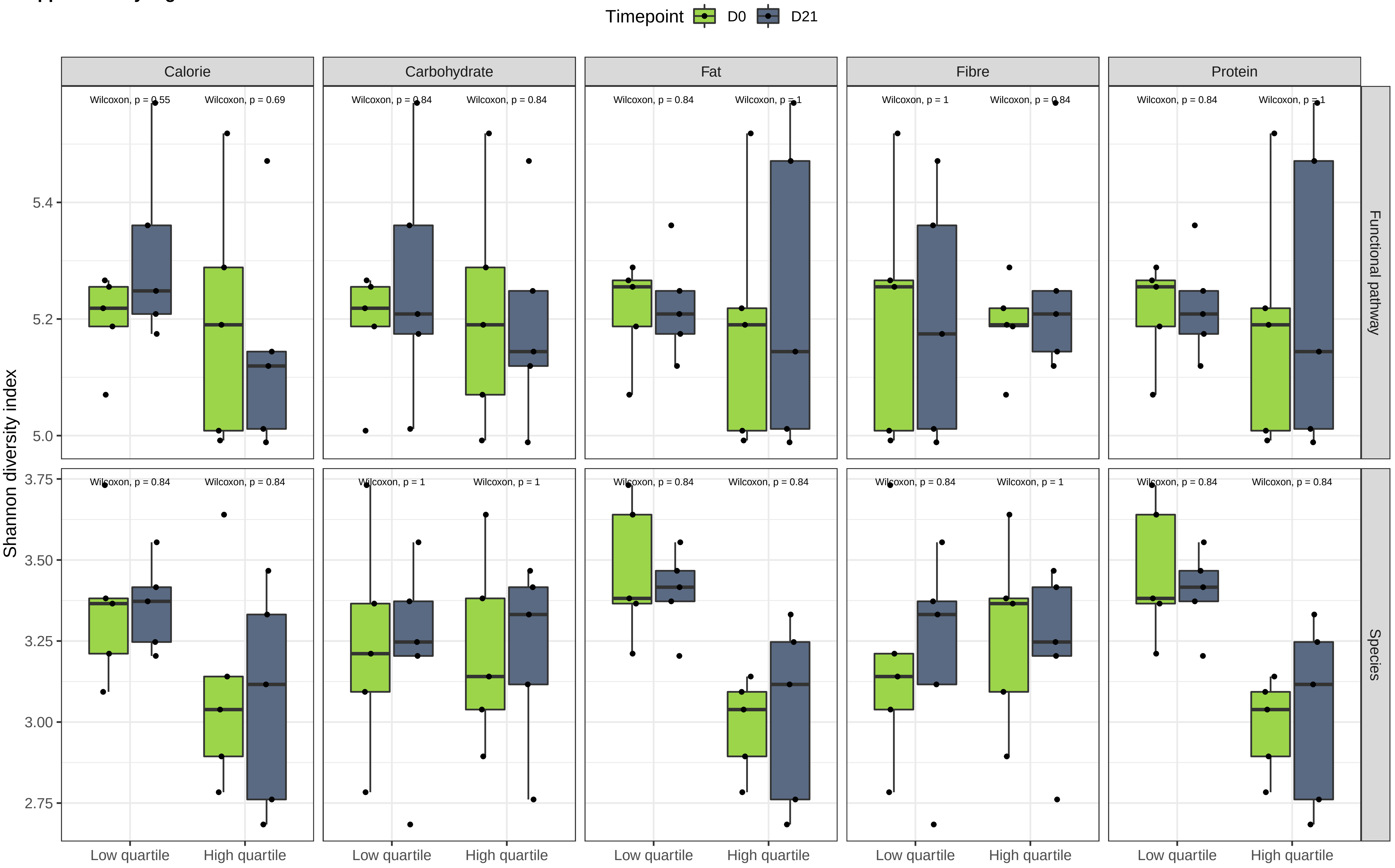

Supplementary Figure 4: Shannon diversity of subjects based on levels of nutrients consumed

Supplementary Figure 5

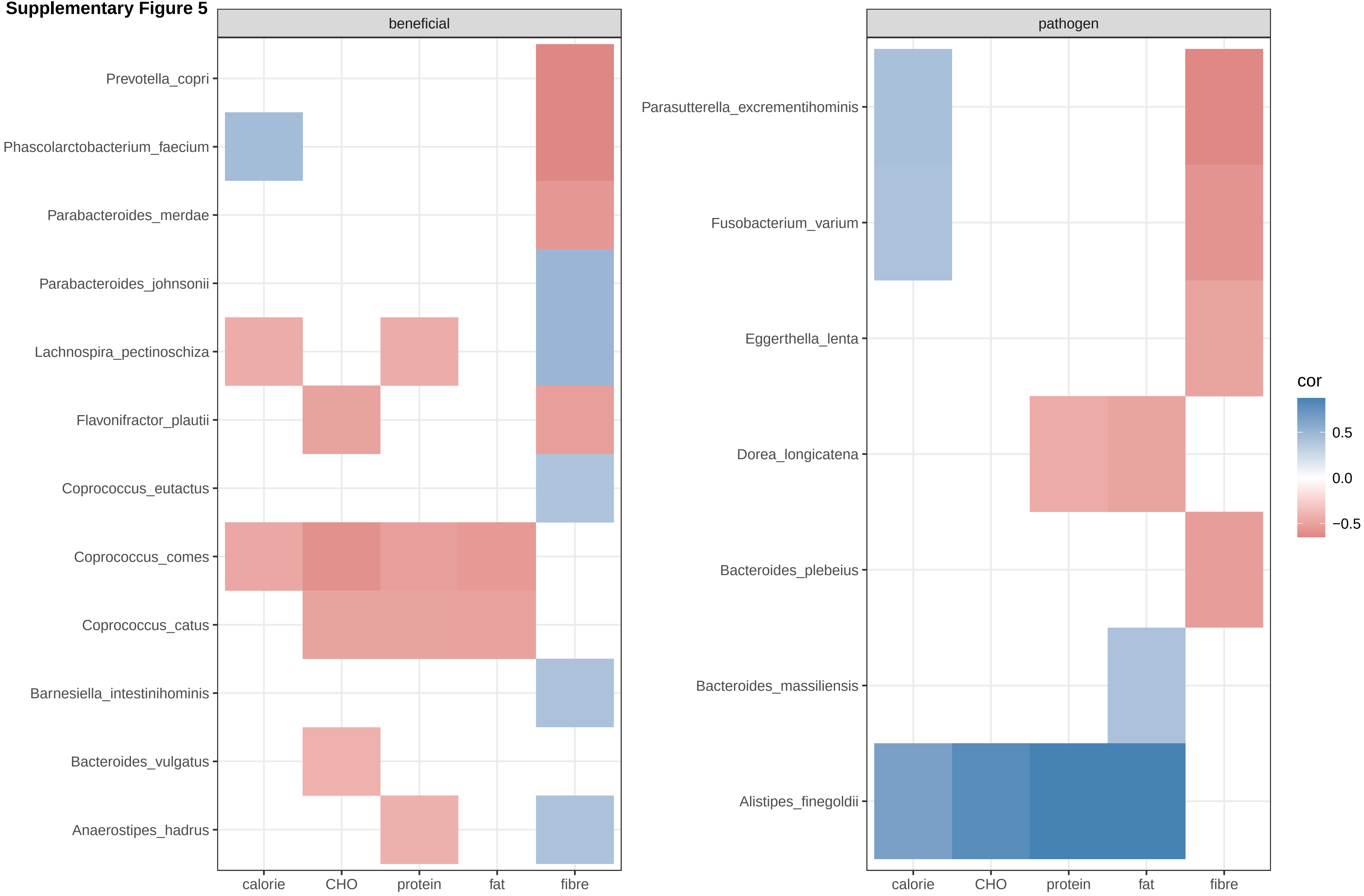

Supplementary Figure 5: Correlation between reported beneficial and pathogenic species with nutrient consumed
